# Differential roles of TNFα-TNFR1 and TNFα-TNFR2 in the differentiation and function of CD4^+^Foxp3^+^ induced Treg cells in vitro and in vivo periphery in autoimmune diseases

**DOI:** 10.1038/s41419-018-1266-6

**Published:** 2019-01-10

**Authors:** Sujuan Yang, Chichu Xie, Ye Chen, Julie Wang, Xiaoqing Chen, Zhengqi Lu, Rayford R. June, Song Guo Zheng

**Affiliations:** 1 Department of Clinical immunology, Third Hospital at Sun Yat-sen University, 510630 Guangzhou, China; 2 Division of Rheumatology, Penn State College of Medicine and Milton S. Hershey Medical Center, Hershey, 17033 USA; 3 Division of Neurology, Third Hospital at Sun Yat-sen University, 510630 Guangzhou, China

## Abstract

Tumor Necrosis Factor (TNF) α is a multifunctional cytokine with pro-inflammatory and anti-inflammatory characteristics. Increasing evidence suggests that thymus-derived, natural regulatory T cells (nTreg) express a remarkably high level of TNF Receptor 2 (TNFR2) and TNFα modulates the number or function of nTreg via TNFR2 in autoimmune diseases. Nonetheless, Treg cells consist of at least nTreg and iTreg that are induced in the periphery or in vitro and two subsets may have different biological characteristics. However, the role of TNF-TNFR signaling in development and function of these iTreg cells is less clear. In this study, we systemically studied the effect of TNFα and its receptor signals on iTreg differentiation, proliferation, and function in vitro and in vivo. We further investigated the expression and requirement of TNFR1 or TNFR2 expression on iTreg by utilizing TNFR1^−/−^ and TNFR2^−/−^ mice. We found that exogenous TNFα facilitated iTreg differentiation and function in vitro. TNFR2 deficiency hampered iTreg differentiation, proliferation, and function, while TNFR1 deficiency decreased the differentiation of inflammatory T cells such as Th1 and Th17 cells but maintained the regulatory capabilities of iTreg both in vitro and in vivo. Using colitis model, we also revealed TNFR2 but not TNFR1 deficiency compromised the iTreg functionality. Interestingly, inflammation affects TNFR expression on nTreg but not iTreg subset. Our results demonstrate that exogenous TNFα may enhance the differentiation and function of iTreg via TNFR2 signaling. The expression of TNFR2 on Treg might be downregulated in some autoimmune diseases, accompanied by an increased level of TNFR1. Thus, TNFR2 agonists or TNFR1-specific antagonists hold a potential promise for clinical application in treating patients with autoimmune diseases.

## Introduction

Tumor Necrosis Factor α (TNFα) plays critical roles in the pathogenesis of inflammatory diseases. TNFα inhibitor therapy is important to treat many autoimmune diseases. Nonetheless, at least 50% of patients with inflammatory diseases are less effective. We hypothesize that TNFα may have a different functional effect on T cells via their respective receptors.

TNFα exerts its function via two receptors, TNFR1 and TNFR2. TNFR1 is ubiquitously expressed on nearly all cells, while TNFR2 is restricted to T lymphocytes and other cells^1,2^. Regulatory T cells (Treg) are the population of prototypic immunosuppressive T cells that terminate excessive autoimmune responses and maintain immune homeostasis^3,4^. The imbalance of the number and/or function of Treg and pathogenesis cells can lead to a wide variety of human autoimmune diseases, including multiple sclerosis (MS), rheumatoid arthritis (RA), and type I diabetes^5,6^.

The role of TNFα in affecting Treg has been a hotspot although the studies are still controversial in the field. Some investigations demonstrated that the stimulation of TNFα enhanced Treg proliferation and suppressive capabilities^7,8^. They also found that Treg expressed a remarkably higher level of TNFR2 than effector T cells (Teffs)^7^ and TNFR2-expressed Treg exhibited optimum suppressive function^9–11^. In contrast, some investigators reported that TNFα decreased the suppressive function of Treg^12–14^.

It has been recognized that Treg consist of two identified subsets: thymic derived natural Treg (nTreg) and induced Treg (iTreg) generated in the periphery from CD4^+^CD25^−^T cells or induced from naive CD4^+^ T cells in vitro^15–17^. We and other researchers have reported that in some autoimmune diseases, nTreg may lose Foxp3 expression and convert to T helper cells, such as Th1, Th17 cells^18,19^. Conversely, iTreg may have a different biological feature and be resistant to phenotypic plasticity^20–22^. However, the effect of TNFα on iTreg has not been well delineated previously. We investigated the effects of exogenous TNFα and TNFR on the differentiation, proliferation, and suppressive function of iTreg, as well as T helper cells.

## Results

### rmTNFα facilitates the differentiation of iTreg and enhances its stability in vitro

**Fig. 1:**
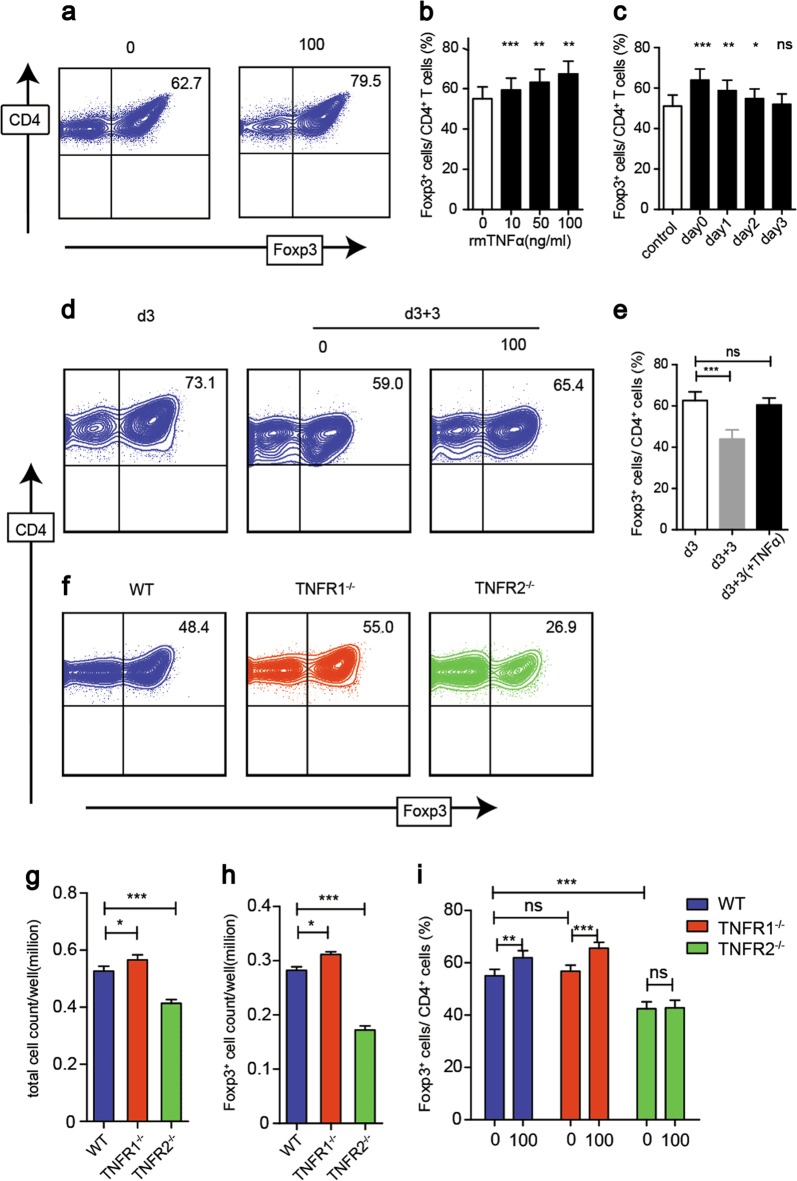
rmTNFα increases iTreg differentiation via TNFR2 in vitro. **a**, **b** Naive CD4^+^ T cells were induced into iTreg with different doses of rmTNFα for three days. The percentages of Foxp3^+^ T cells were determined. **c** During iTreg induction, the same dose of rmTNFα was added on day 0, 1, 2, or 3. All the cells were harvested after 4 days. The percentages of Foxp3^+^ T cells were determined. **d**, **e** iTreg induced for three days and reseeded with or without rmTNFα for another three days. The percentages of Foxp3^+^ T cells were determined. **f**, **g**, **h** Naive CD4^+^ T cells isolated from WT, TNFR1^−/−^, and TNFR2^−/−^ mice were induced to iTreg. The percentages of Foxp3^+^ T cells, the total cell numbers and Foxp3^+^ cell numbers were determined, respectively. **i** Naive CD4^+^ T cells isolated from WT, TNFR1^−/−^, and TNFR2^−/−^ mice were induced to iTreg with or without rmTNFα for three days. The percentages of Foxp3^+^ T cells were determined. *P ≤ 0.05; **P ≤ 0.01; ***P ≤ 0.001, error bars denote SD. Representative result is from five independent experiments

To investigate whether TNFα impacts iTreg differentiation, naive CD4^+^ T cells from WT mice were induced into iTreg as previously reported with or without recombinant mouse TNFα (rmTNFα)^22^. Our results showed that rmTNFα stimulation markedly increased iTreg differentiation in a dose-dependent manner (Fig. 1a, b). Additionally, we observed that TNFα exposure did not affect the viability of Treg, even as high as 100 ng/ml (Sups 1. a, b). To exclude the possibility that the augment of Foxp3 expression was caused by the expansion of iTreg that had been previously induced, we added rmTNFα at different time points during iTreg differentiation periods. We found that the earlier rmTNFα was added in, the higher Foxp3 was expressed on CD4^+^ T cells (Fig. 1c). Moreover, we also induced naive CD4^+^ T cells into Th1, Th17 cells in the absence or presence of rmTNFα. We found that the stimulation of rmTNFα did not significantly change Th1 and Th17 cells differentiation in vitro (Sups 1. c, d, g, h).

Next, we evaluated the effect of TNFα on the stability of iTreg. We found that in the absence of rmTNFα, Foxp3 expression of iTreg gradually decimated, while rmTNFα exposure led to maintaining Foxp3 expression and cell viability (Fig. 1d, e). These results suggest that TNFα facilitates iTreg differentiation and enhances its stability.

### TNFR2 promotes iTreg differentiation while TNFR1 mediates the differentiation of inflammatory T cells in vitro

To further investigate whether TNFα mediates the enhancement of iTreg differentiation through TNFR1 or TNFR2, we used TNFR1^−/−^ or TNFR2^−/−^ mice. Our results showed that TNFR1^−/−^ naive CD4^+^ T cells can differentiate to iTreg comparably with those from WT mice. Nonetheless, TNFR2 deficiency dramatically hampered their ability to differentiate into iTreg (Fig. 1f, i). The exposure of rmTNFα promoted WT and TNFR1^−/−^ not TNFR2^−/−^ naive CD4^+^ T cells to differentiate into iTreg (Fig. 1i). Furthermore, the numbers of total cells and Foxp3^+^ cells in TNFR2^−/−^ group were both significantly less than WT group (Fig. 1g, h). It raises a possibility that TNFR2 signaling plays an important role in iTreg differentiation.

We also performed the induction of Th1 and Th17 differentiation on naive CD4^+^ T cells derived from WT, TNFR1^−/−^, and TNFR2^−/−^ mice. Notably, fewer IFN-γ^+^ cells and IL-17^+^ cells were detected in TNFR1^−/−^ cells than in WT cells whereas TNFR2^−/−^ naive CD4^+^ T cells can differentiate into Th1 or Th17 cells at a similar proportion with WT cells (Sups 1. e, f, i, j). It suggests that TNFR1 and its downstream signaling pathways play a vital role in the Th1 and Th17 cell differentiation.

### TNFR2 promotes Treg differentiation while TNFR1 mediates the differentiation of inflammatory T cells in vivo

**Fig. 2:**
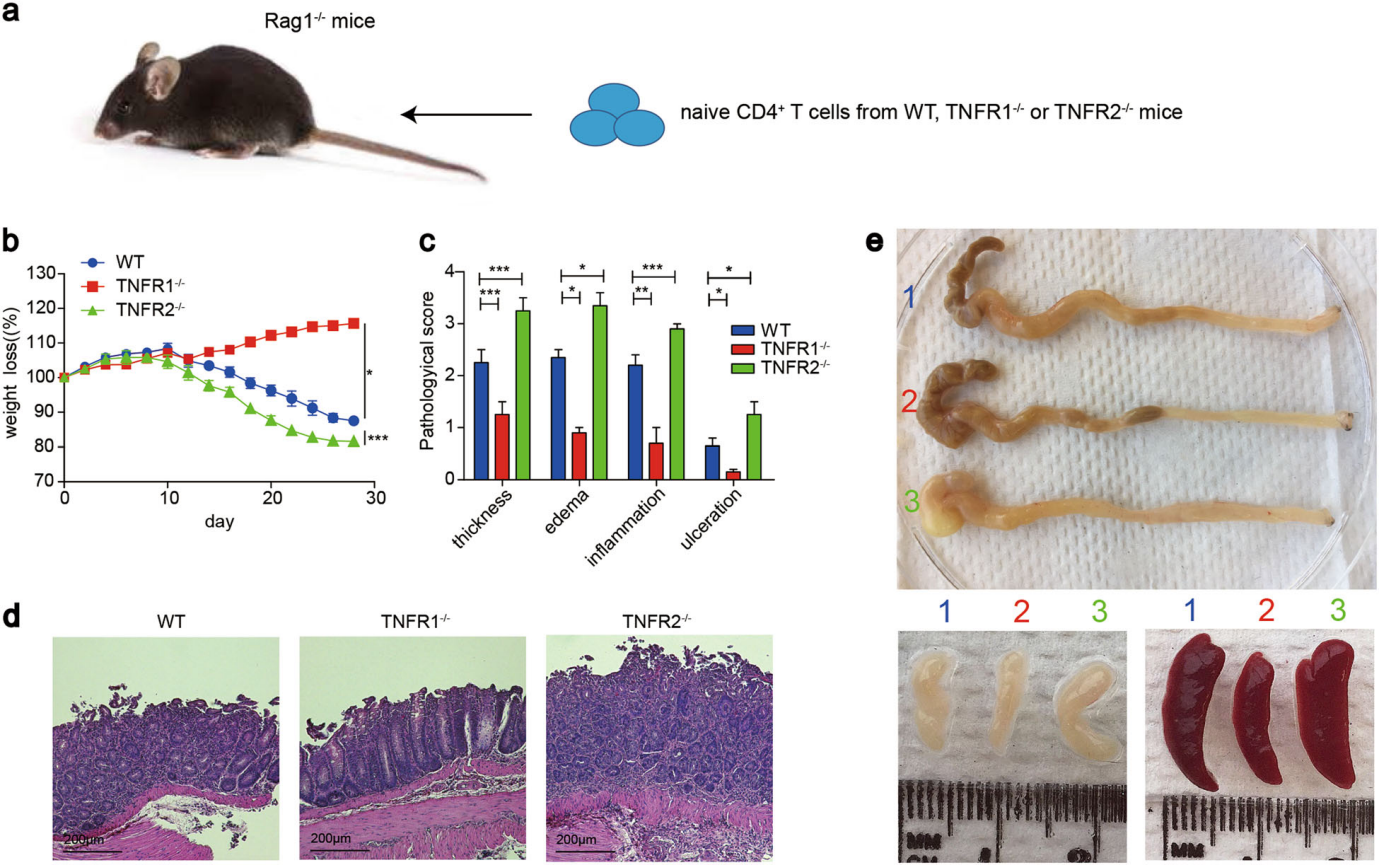
TNFR2 gene knockout enhanced inflammatory responses and TNFR1 gene knockout was resistant to the development of colitis. **a** Naive CD4^+^ T cells isolated from WT, TNFR1^−/−^, and TNFR2^−/−^ mice were injected into Rag1^−/−^ mice intraperitoneally. **b** Weights of the recipient mice were monitored after the cell transfer. **c**, **d** The colon was subjected to staining with H&E (×40, scale bars = 200 μm), and the development of colitis was evaluated blindly by two pathologists. **e** The morphology and size of the colon, spleen and mLNs from the recipient mice were compared. No.1 was to WT group; No.2 was to TNFR1^−/−^ group; No.3 was to TNFR2^−/−^ group. *P ≤ 0.05; **P ≤ 0.01; ***P ≤ 0.001, error bars denote SD. Representative result is from six independent experiments

**Fig. 3:**
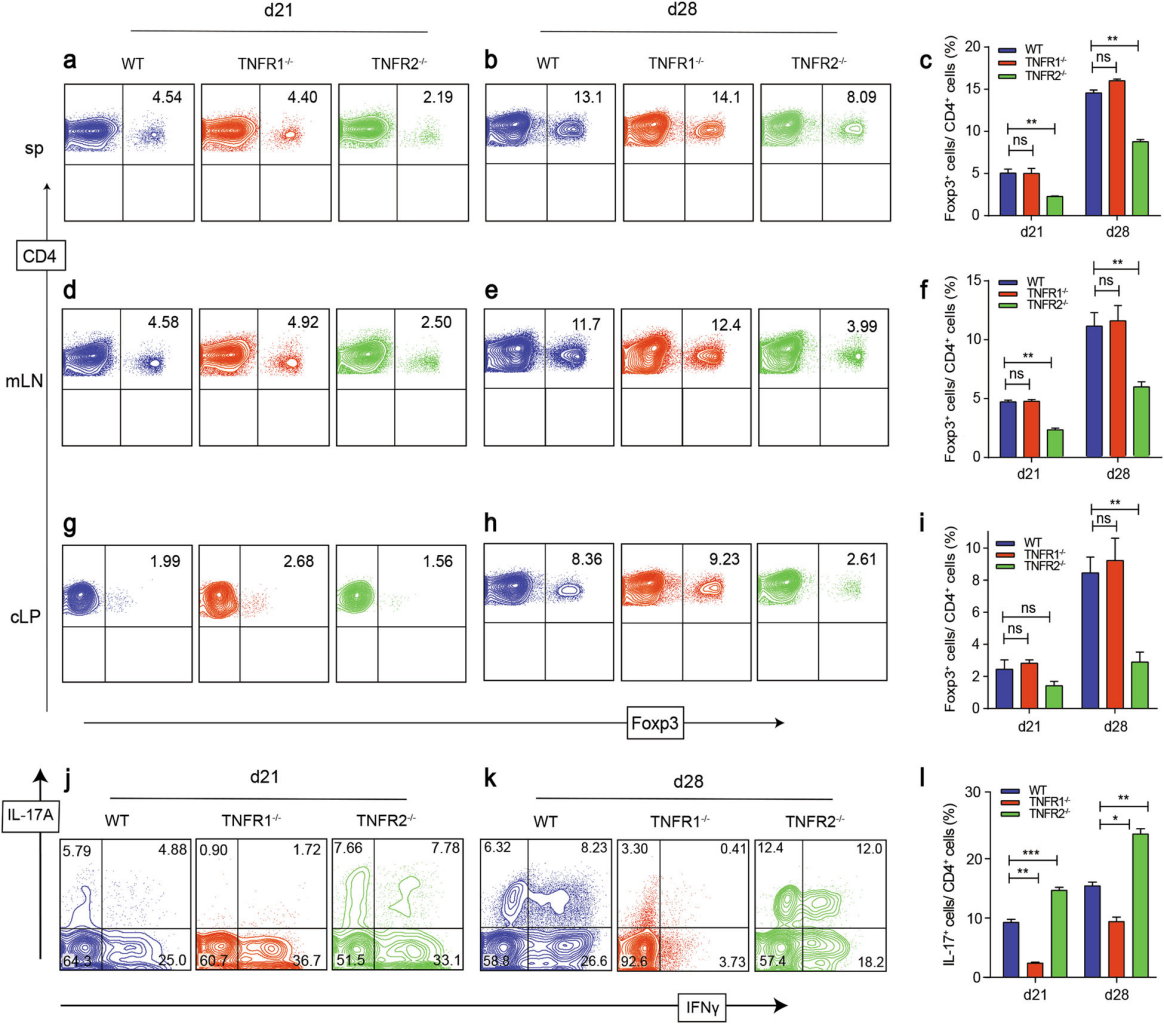
The effect of TNFR1 and TNFR2 on iTreg, Th1, Th17 cell differentiation in vivo. **a** Naive CD4^+^ T cells isolated from WT, TNFR1^−/−^, and TNFR2^−/−^ mice were injected into Rag1^−/−^ mice intraperitoneally. **a–i** 21 days and 28 days after the transfer, spleens, mLNs, and cLP were harvested and the proportions of Foxp3^+^ T cells were determined. **j–l** The proportions of IL-17A^+^ or IFN-γ^+^ T cells in cLP were determined. *P ≤ 0.05; **P ≤ 0.01; ***P ≤ 0.001, error bars denote SD. Representative data is from six independent experiments

To examine the role of TNFR1 and TNFR2 in Treg differentiation in vivo, we used a standard in vivo protocol we recently established (Fig. 2a)^23^. We found that the Rag1^−/−^ mice that received TNFR1^−/−^ naive CD4^+^ T cells slowly gained weight and only caused slight colitis (Fig. 2b), while TNFR2^−/−^ naive CD4^+^ T cells caused the most severe weight loss, and H&E staining showed the most severe inflammatory cells infiltration, ulcer, edema, and bowel wall thickening among the three groups (Fig. 2c, d). We also observed the most severe bowel wall edema and lack of formed feces, spleen, and mLNs enlargement in TNFR2^−/−^ group (Fig. 2e). Consistent with these results, on day 21, in spleens and mLNs, the proportion of Foxp3^+^ T cells in TNFR1^−/−^ group was comparable with WT group. However, TNFR2^−/−^ naive CD4^+^ T cells failed to differentiate to Treg (Fig. 3a, d). A similar result was observed in cLP (Fig. 3g). On day 28, in WT and TNFR1^−/−^ groups, the proportion of Foxp3^+^ T cells increased by 3–4 folds both in spleens and mLNs, even by 4–5 folds in cLP. However,TNFR2^−/−^ Treg exhibited a hampered proliferation ability (Fig. 3a–i).

In cLP, the frequency of Th17 cells dramatically increased in TNFR2^−/−^ group, while TNFR1^−/−^ naive CD4^+^ T cells were partially resistant to Th17 differentiation (Fig. 3j–l). A similar result was observed in spleen and mLNs (Sups 2. a–f). However, Th1 cell differentiation did not show a uniformed significant difference among spleen, mLNs, and cLP, which indicates that the effect of TNFR signal pathways on Th1 differentiation in vivo might be more complicated.

**Fig. 4:**
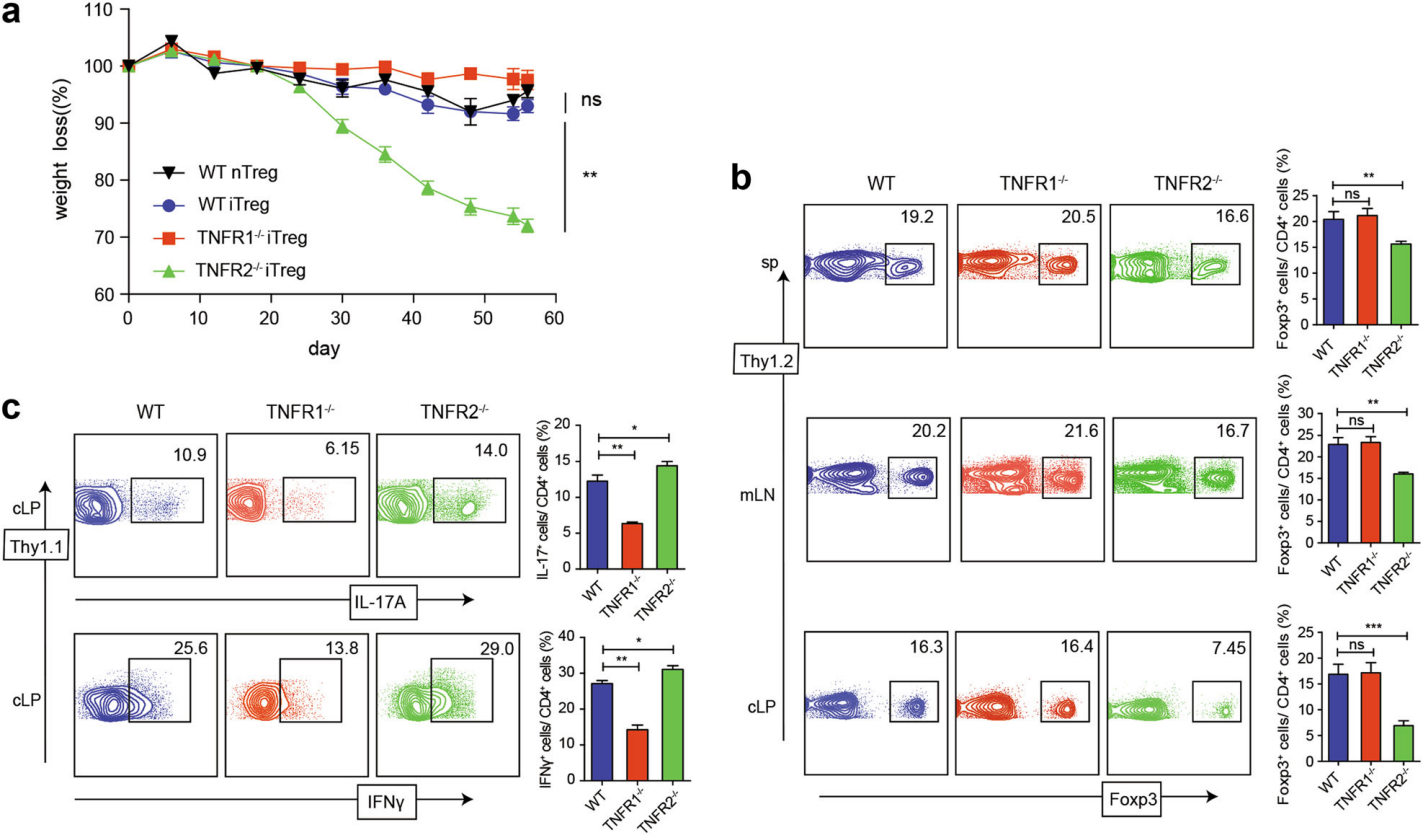
The effect of TNFR1 and TNFR2 on iTreg stability and function in vivo. iTreg were induced from three strains (Thy1.2^+^) as above protocols. 3 days later, these cells (0.6 **×** 10^6^) were i.v. injected into Rag1^−/−^ mice that has received WT Thy1.1^+^ naive CD4^+^ T cells (0.6 **×** 10^6^) through i.p. injection on the same day. **a** Weights of the recipient mice were monitored after the cell transfer. **b** 56 days after the transfer, spleens, mLNs, and cLP were harvested and the proportions of Foxp3^+^ T cells gated on Thy1.2^+^ cells were determined. **c** The proportions of IL-17A^+^ or IFN-γ^+^ T cells gated on Thy1.1^+^ cells in cLP were determined. *P ≤ 0.05; **P ≤ 0.01; ***P ≤ 0.001, error bars denote SD. Representative data is from six similar independent experiments

As TNFRs can be also expressed on CD4^+^Foxp3^−^ T cells, the colitis model induced by naive CD4^+^ T cells could be a misleading. To clarify this concern, we have induced iTreg with naive CD4^+^ T cells from Thy1.2^+^ WT, TNFR1^−/−^, and TNFR2^−/−^ strains and injected them into Rag1^−/−^ mice with WT Thy1.1^+^ naive CD4^+^ cells simultaneously. On day 56, we found that the Rag1^−/−^ mice that had received either WT nTreg or WT iTreg had a similarly disease protection, suggesting both nTreg and iTreg have a similar suppressive effect on colitis. Interestingly, compared to WT iTreg, TNFR1^−/−^ iTreg infusion almost completely maintained the weight during colitis, conversely, TNFR2^−/−^ iTreg infusion failed to protect the mice from colitis and resulted in severe weight loss (Fig. 4a). In line with these results, the proportion of Foxp3^+^ T cells gated on Thy1.2^+^ cells in TNFR1^−/−^ iTreg infusion group was comparable with WT group in spleen, mLNs, and cLP. However, TNFR2^−/−^ Treg infusion failed to maintain their Foxp3 expression (Fig. 4b). In cLP, the frequency of Th1 and Th17 cells gated on Thy1.1^+^ cells was significantly greater in TNFR2^−/−^ iTreg infusion than in TNFR1−/− or WT iTreg infusion groups (Fig. 4c). These results further validate that TNF-TNFR2 signal promotes iTreg differentiation, stability, and functionality.

**Fig. 5:**
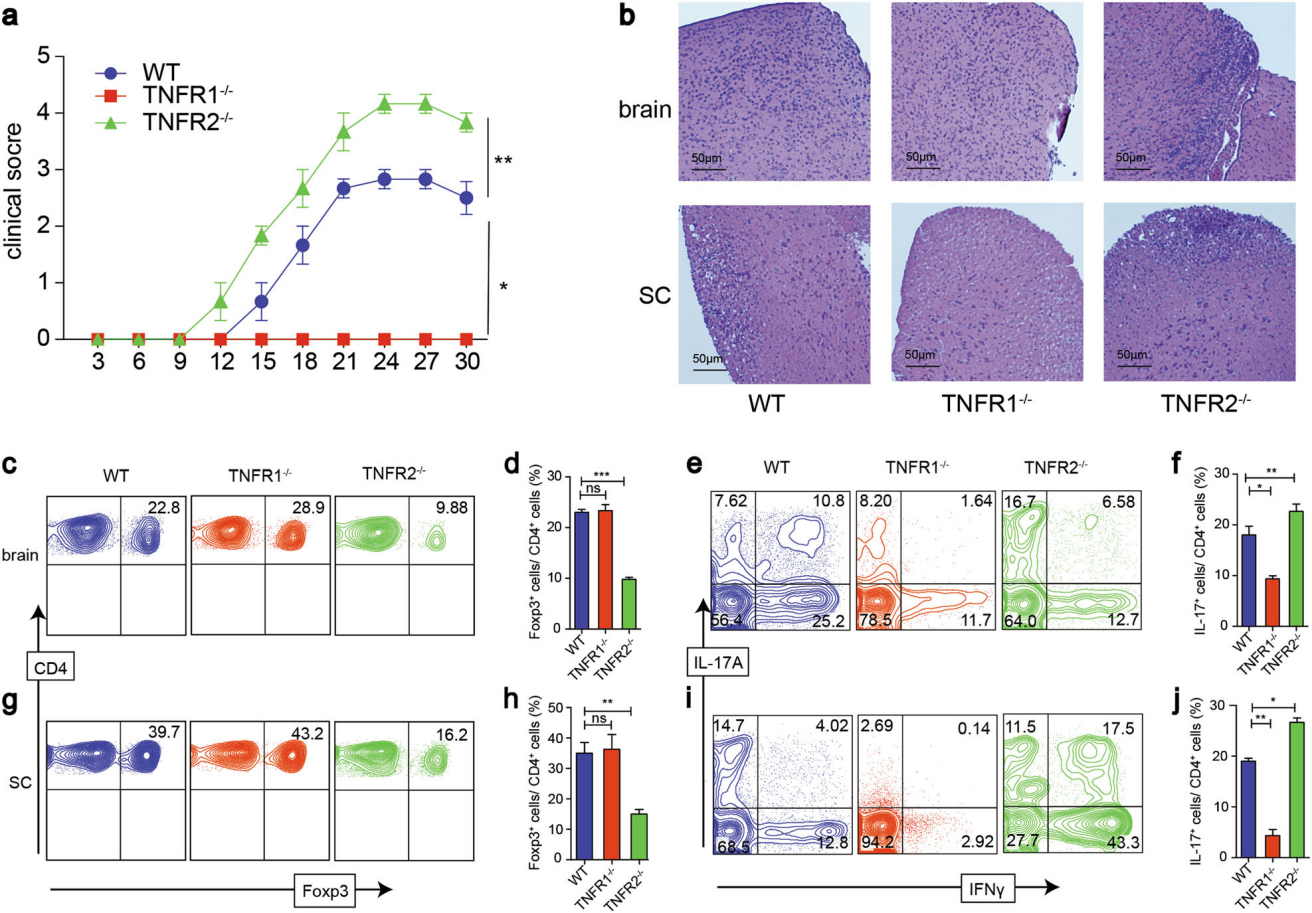
The effect of TNFR1 and TNFR2 on Treg, Th1, Th17 cells in EAE mouse model. **a** EAE model were induced on WT, TNFR1^−/−^, and TNFR2^−/−^ Foxp3-reporter mice, the clinical symptom scores were evaluated blindly. **b** 30 days after the first immunization, part of the brain and spinal cord were stained with H&E (×100, scale bars = 50 μm). **c**, **d**, **g**, **h** The proportions of Treg cells in brain and spinal cord (SC) were detected by FACS. **e**, **f**, **i**, **j** The proportions of Th1 and Th17 cells in brain and spinal cord were detected by FACS. *P ≤ 0.05; **P ≤ 0.01; ***P ≤ 0.001, error bars denote SD. Representative data is from six independent experiments

We also induced the experimental autoimmune encephalomyelitis (EAE), an animal model of patients with MS, on WT, TNFR1^−/−^, and TNFR2^−/−^ mice to evaluate the role of TNFR in the pathogenesis of autoimmune diseases. Consistent with a previous report^24^, we found that TNFR1^−/−^ mice were completely resistant to the inflammatory effect without any EAE symptoms, while, TNFR2^−/−^ mice had an early onset of inflammation and developed severe disease (Fig. 5a). The brain and spinal cord (SC) of TNFR2^−/−^ mice displayed more severe cellular infiltration, particularly in myelinated regions and tissue damage than WT mice (Fig. 5b). Furthermore, the proportions of Treg in TNFR2^−/−^ mice were markedly lower than that in WT mice (Fig. 5c, d, g, h). However, the proportion of Th17 cells was the highest in TNFR2^−/−^ mice, the proportions of Th1 and Th17 cells in TNFR1^−/−^ mice were the least among the three groups (Fig. 5e, f, i, j). These results further document that TNFα via TNFR1 promotes inflammation and via TNFR2 exerts immunoregulation.

### TNFR2 but not TNFR1 promotes Treg proliferation in vitro and in vivo

**Fig. 6:**
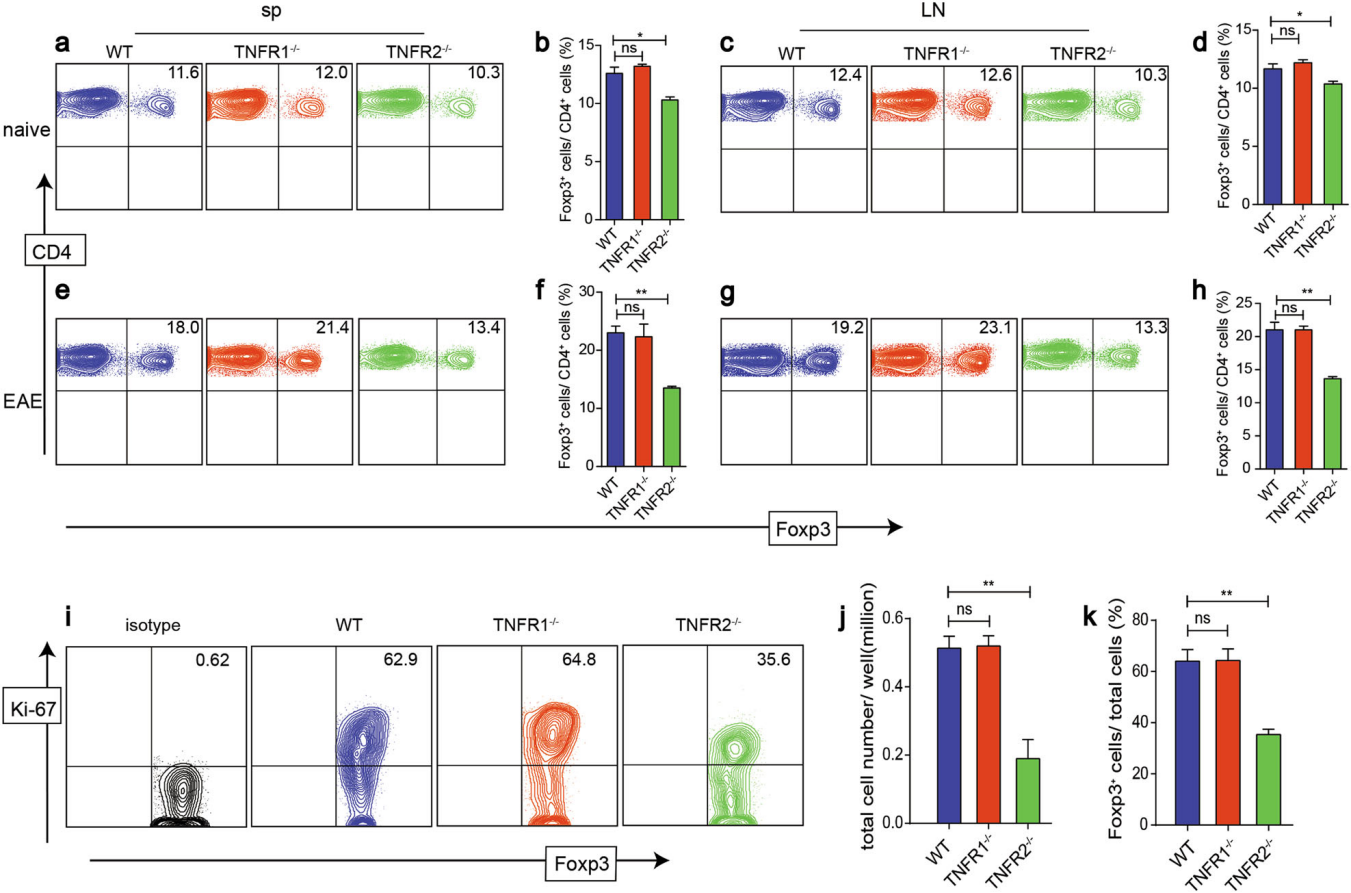
TNFR2 is a critical signaling for Treg expansion following EAE induction and in vitro. **a–d** We detected the proportions of Treg in spleen and LNs from naive WT, TNFR1^−/−^, and TNFR2^−/−^mice. **e–h** 30 days after immunized with MOG, the proportion of Treg in spleen and LNs from the three groups were detected. **i–k** Treg isolated from LNs of WT, TNFR1^−/−^, and TNFR2^−/−^ mice were expanded in vitro. Ki-67 was detected after 3 day culture. *P ≤ 0.05; **P ≤ 0.01, error bars denote SD. Representative data is from six independent experiments

To determine whether TNFR2 deficiency hampers Treg expansion, we evaluated the baseline levels of Foxp3^+^ cells in naive WT, TNFR1^−/−^, and TNFR2^−/−^ mice. As expected, the proportion of Treg in TNFR2^−/−^ mice was marginally lower than those in the spleen and LNs of WT and TNFR1^−/−^ mice (Fig. 6a–d). However, after the immunization, Treg percentage increased strikingly by two folds in spleen and LNs from WT and TNFR1^−/−^ mice, while in TNFR2^−/−^ mice, the increase of Treg population was hardly detected (Fig. 6e–h). We further isolated Treg from WT, TNFR1^−/−^, and TNFR2^−/−^ mice and expanded in vitro. We found that TNFR2^−/−^ Treg expressed the lowest level of Ki-67 (Fig. 6i–k). Furthermore, the numbers of total cells and Foxp3^+^ cells in TNFR2^−/−^ group were both significantly less than WT group.

### rmTNFα via TNFR2 enhances the suppressive function of iTreg in vitro

**Fig. 7:**
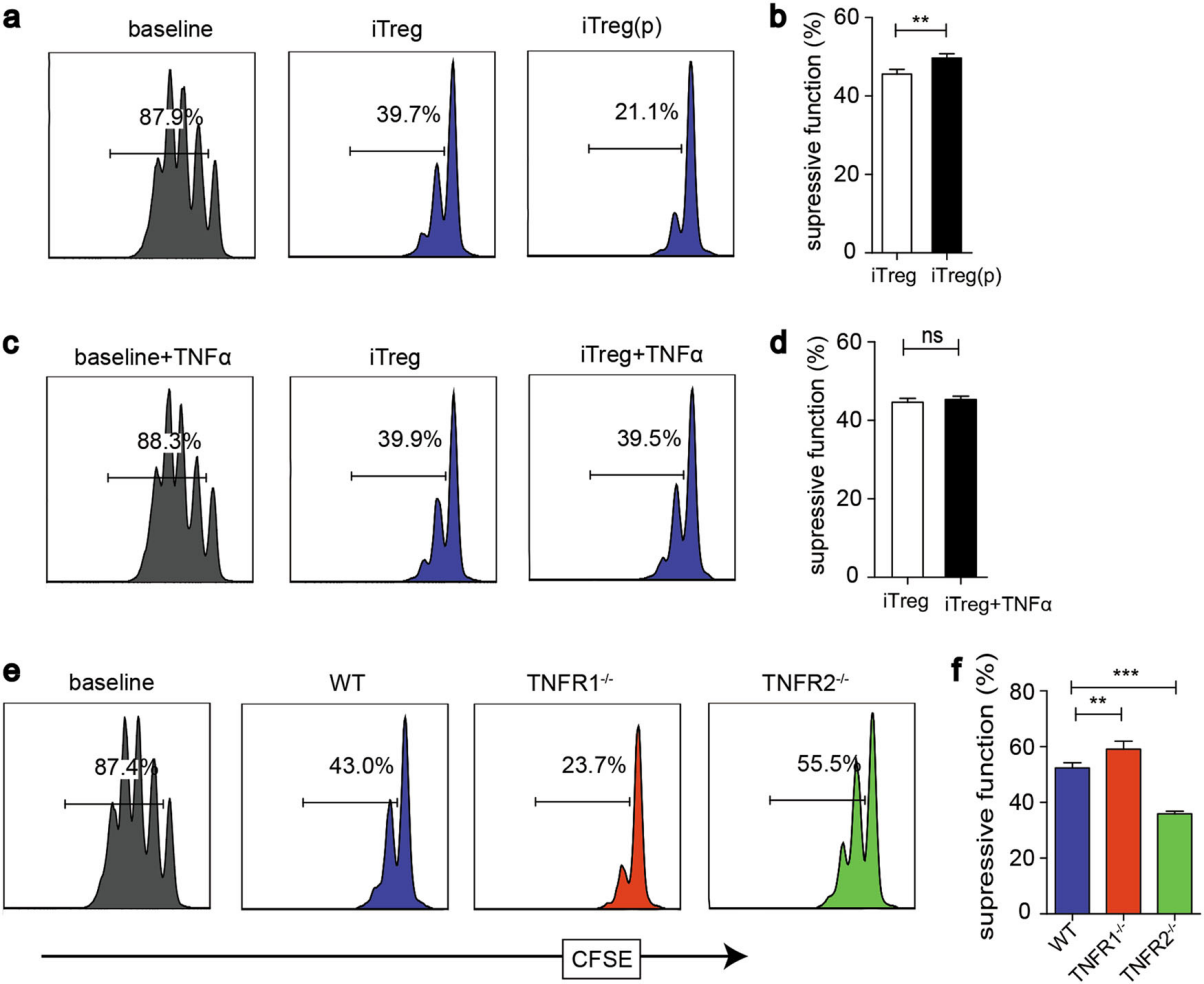
rmTNFα enhances iTreg impressive function via TNFR2 in vitro and TNFR2 mediated the Suppressive capability of iTreg in vivo. **a**, **b** iTreg were induced with or without rmTNFα. After 3 days, iTreg were co-cultured with CFSE-labeled Teffs. **c**, **d** iTreg were induced without rmTNFα, and then were co-cultured with Teffs with or without rmTNFα in the culture medium. **e**, **f** iTreg induced from WT, TNFR1^−/−^, and TNFR2^−/−^ mice were co-cultured with Teffs. **P ≤ 0.01; ***P ≤ 0.001, error bars denote SD. Representative data is from six independent experiments

We also investigated the contribution of TNFα to iTreg suppressive function in vitro. The CFSE assay showed considerable proliferation of CD8^+^ T cells at baseline. With the stimulation of rmTNFα, the replications of Teffs were similar to those in the absence of TNFα, suggesting that this dose of rmTNFα did not significantly enhance the proliferation of Teffs. iTreg pretreated with rmTNFα exhibited superior suppressive effect to those without the pretreatment (Fig. 7a, b). Furthermore, we induced iTreg without rmTNFα, and compared iTreg function on Teffs in the culture condition with or without rmTNFα. We found that under the exposure of rmTNFα, iTreg showed similar excellent suppression on Teffs proliferation when compared to those without TNFα in the medium (Fig. 7c, d). To determine the contribution of TNFR to the suppressive capability of iTreg, we compared the suppressive function in vitro of iTreg generated from WT, TNFR1^−/−^, and TNFR2^−/−^ mice. As expected, TNFR2^−/−^ iTreg exhibited impaired suppressive activity, while the suppressive activity of TNFR1^−/−^ iTreg is slightly superior to WT iTreg (Fig. 7e, f).

### TNFR2 mediates the suppressive capability in vivo of iTreg

**Fig. 8:**
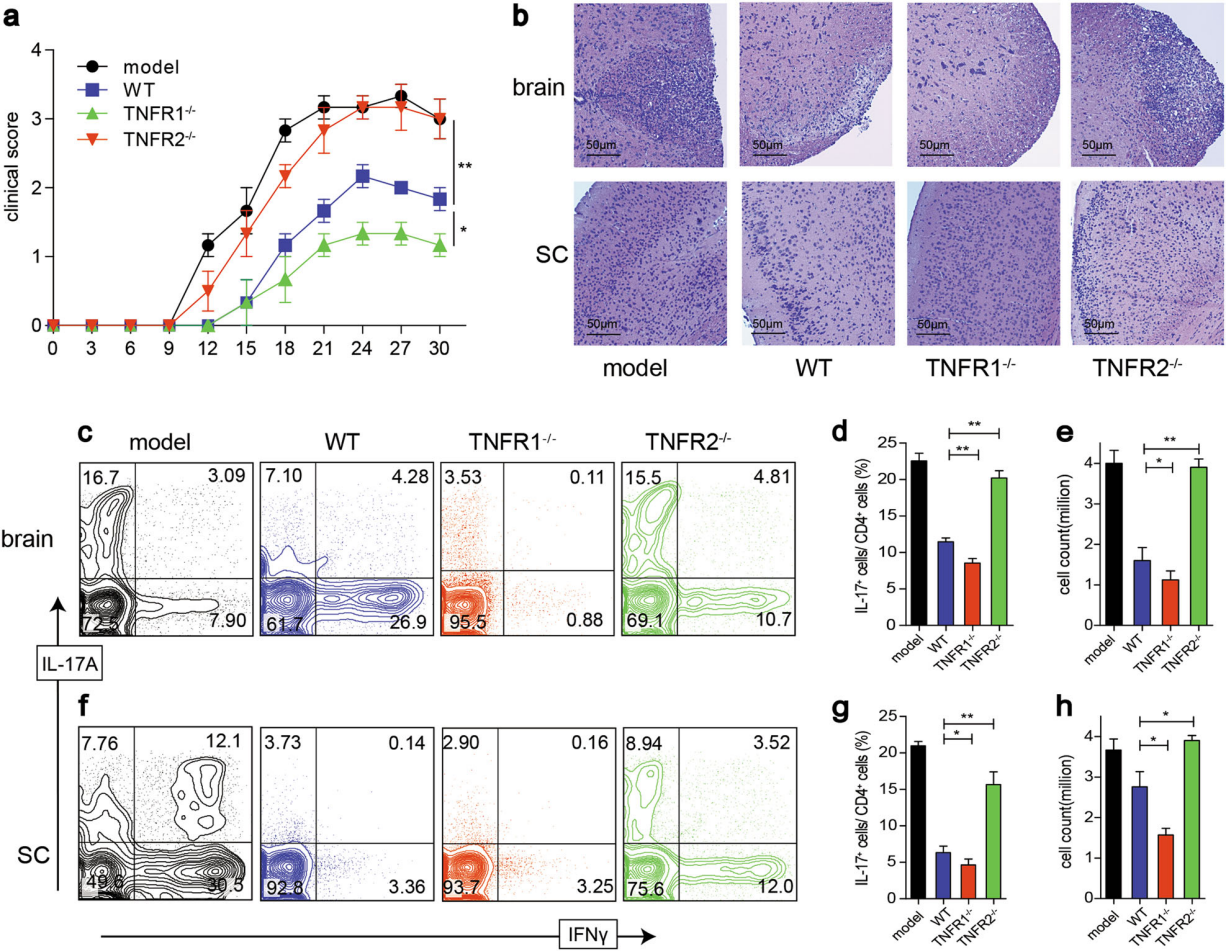
rmTNFα enhances iTreg suppressive function via TNFR2 in vitro and TNFR2 mediated the suppressive capability of iTreg in vivo. **a** EAE was induced on WT mice. 9 days after the first immunization, iTreg derived from WT, TNFR1^−/−^, and TNFR2^−/−^ mice were adoptively transferred into EAE model, respectively. The clinical scores were monitored. **b** 30 days after the first immunization, the brain and spinal cord (SC) were subjected to H&E staining (×100, scale bars = 50 μm). Foxp3, IL-17A, and IFN-γ expression in brains (**c**–**e**) and SC (**f**–**h**) were detected. *P ≤ 0.05; **P ≤ 0.01, error bars denote SD. Representative data is from six independent experiments

We also investigated the therapeutic effect of iTreg derived from WT, TNFR1^−/−^, and TNFR2^−/−^ mice on the EAE model induced in WT mice. We observed that WT iTreg exerted optimal inhibitory function on the treatment of EAE, delayed onset, and ameliorated the symptoms (Fig. 8a). Moreover, TNFR1^−/−^ iTreg marginally exhibited superior suppressive function to WT iTreg, which largely diminished the autoimmune responses in the brain and SC, little cellular infiltration was detected (Fig. 8b). However, TNFR2^−/−^ impaired suppressive function of iTreg, which failed to ameliorate the disease, Th17 cells increased dramatically in brain and SC, where inflammatory cells were massively accumulated that is similar with colitis model (Fig. 8c–h).

### TNFR1 and TNFR2 expression is disordered on CD4^+^ nTreg, while CD4^+^ iTreg maintained their TNFRs expression in EAE onset mice when compared with naive mice

We also detected whether iTreg express a high level of TNFR2 like nTreg and compared the expression of TNFR1 and TNFR2 on WT, TNFR1^−/−^, and TNFR2^−/−^ iTreg. Our results demonstrated that naive CD4^+^ T cells expressed a low level of TNFR2. After iTreg induction, TNFR2 expression on T cells was elevated, and those on TNFR1^−/−^ iTreg was significantly higher than those on WT iTreg. Vice versa, TNFR2^−/−^ iTreg expressed a higher level of TNFR1 than WT iTreg (Sup 3. a).

We also detected the expression of other phenotypic markers on iTreg. The results showed that CD103 expression was upregulated on TNFR1^−/−^ iTreg, and downregulated on TNFR2^−/−^ iTreg (Sup 3. b). CD103 has been well explored to define the most potent suppressive subset of Treg^25^. Previous reports have demonstrated an interaction between TNFR2 and CD103^26^. This suggests TNFR2 signaling pathway might synergistically interact with those cells expressing CD103.

To determine if TNFR1 and TNFR2 expression is different on nTreg from on iTreg when the mice suffer from autoimmune diseases, we also analyzed their expression on nTreg of WT, TNFR1^−/−^, and TNFR2^−/−^ mice and compared the two TNFRs on naive and disease mice. Our results showed that TNFR2 expression on TNFR1^−/−^ nTreg was significantly higher than those on WT nTreg. Conversely, TNFR2^−/−^ nTreg expressed a higher level of TNFR1 than WT nTreg in naive mice (Sups 4. a, c), both are similar with iTreg subset (Sups 5. a, c). When the mice suffered from EAE induction, nTreg expressed an elevated level of TNFR1, especially higher in TNFR2^−/−^ mice, and a dramatically reduced level of TNFR2 both in WT and TNFR1^−/−^ mice (Sups 4). Surprisingly, we found that iTreg maintained their TNFR1 and TNFR2 expression in inflammation condition of colitis model (Sups 5).

## Discussion

Through a series of observations, we have demonstrated that exogenous TNFα might increase iTreg differentiation via TNFR2, which confirms previous reports related to nTreg both in human and mice^7,8^. We have used two sets of animal disease models which demonstrated that TNFR2 deficient mice presented the earliest onset and developed the most severe symptoms of autoimmunity.

When challenged with the induction of EAE, WT, and TNFR1^−/−^ Treg proliferated normally, but TNFR2^−/−^ Treg hardly expanded to inhibit the extensive inflammation. Similarly, TNFR2^−/−^ Treg failed to expand and had poor viability in vitro. Our findings provide compelling evidence that in the context of autoimmunity, TNFR2 is also essential for promoting Treg proliferation.

Our data also demonstrated that TNFα increases iTreg suppressive function and this appears to be dependent upon TNFR2 signal. Additionally, TNFR1^−/−^ iTreg expressed a slightly higher level of TNFR2 than WT iTreg. We propose the possibility that TNFR1 deficiency is secondary to increased TNFR2 signaling, as a result of increased ligand availability to remedy a loss of TNFR1 signaling. According to another study which demonstrated that shedding large amounts of TNFR2 by Treg inhibited the inflammatory action of TNFα^27^, one hypothetical mechanism for this is that TNFR1^−/−^ iTreg increased TNFR2 expression and enhanced the suppressive function by shedding more sTNFR2. TNFR2^−/−^ iTreg cells are not able to shed sTNFR2 and to exert their suppressive function. Our results are also consistent with recent investigations in the type 1 diabetes model on NOD mice, which found that TNFR1 deficiency protected the mice from diabetes by promoting the expansion and function of Treg via TNFR2^28^. Additionally, other have used an acute inflammation model, acute graft-versus-host disease (GVHD) mouse model, to document that TNFα in serum selectively activated Treg and induced Treg proliferation and function without impacting CD4^+^Foxp3^−^ T cells^29^. Similarly, Teff-derived TNFα dominates the enhancement and the defect of TNFα on Teffs or TNFR2 on Tregs was sufficient to completely abolish the Treg suppressive effect on acute GVHD mouse model^30^. Thus, role of TNF-TNFR in Treg can be exerted in both acute and chronic inflammation conditions. Our study has confirmed the influence of TNF/TNFR2 on Tregs, and made a further step on how the two TNFRs impact on Treg proliferation and function, especially Tregs induced in vitro, which may overcome the paucity of nTreg and provide a new tool to optimize Treg cell therapy for autoimmune diseases.

However, one study proposed one mechanism that mTNFα/TNFR2 on CD4^+^ T cells inhibited Th17 cell differentiation by activating the *il-2* promoter and maintaining *il-2* mRNA stability in a Treg-independent manner^31^. Nevertheless, another study showed that the anti-TNF antibody, adalimumab, expanded the pool of Treg and maintained their function via mTNFα/TNFR2 associated with low levels of IL-2 production and subsequently STAT5 activation of Treg^32^. Although the precise TNFR2 signaling pathway remains unclear, it highlights that TNFR2 plays a vital role in Treg proliferation and function and our results support these findings.

However, there are still many unanswered questions about the effects of TNFα on Treg. Previous studies have shown that TNFα downregulates Foxp3 expression and blocks the suppressive function of Treg^33,34^. Moreover, another study showed that TNFα inhibited TGF-β-induced Smad3 phosphorylation and consequently reduced *foxp3* promoter activation and neutralization of TNFα selectively promotes new iTreg differentiation but not nTreg^13^. Coincidentally, it has been indicated that anti-human TNFα Ab (infliximab) increased the iTreg frequency and enhanced their suppressor activity, and remained nTreg defective in RA patients and RA mouse model on human TNF-a transgenic mice^35,36^. TNFR2-specific agonist prolonged survival and reduced GVHD severity in a TNFR2- and Treg cell–dependent manner^37^. However, others demonstrated that nTreg but not iTreg require TNFα signaling for in vivo function^38^. The discrepancy has been suggested to be secondary to the cross-species differences between murine and human T cells, the differences in the sensitivity of the experimental conditions or the differing methodologies employed in each laboratory, the early or late stage on immune responses when TNFα stimulation was administered, even whether nTreg or iTreg are the dominant regulatory population in different autoimmune diseases. However, use of TNFR1 and TNFR2 KO mice in the current study is an advantage to determine the role of TNF-TNFR signal pathway in Treg and Th cell differentiation and function. We believe our results provide update insight into this field and these data are solid given several different approaches and disease models have been used in documenting the role of TNF-TNFR1/2 signals in iTreg differentiation, proliferation, and suppressive function. We have used both iTreg induced ex vivo and Treg differentiated in colitis in RAG1 KO mice. Although one might argue the Treg differentiation in RAG1 KO mice may not belong to iTreg subset, however, these cells have developed in the outside of thymus, it should be different from nTreg and can be considered as an iTreg subset induced in the periphery.

The mechanism of TNFα interacting with Treg remains elusive. It is possible that TNFα and IL-2 are produced by activated T cells, and the synergy of these two cytokines promotes the ability of Treg^8^. Secondly, TGF-β can promote TNFα production and TNFR2 expression on CD4^+^ cells^39^ and may subsequently increase of TNFR2^+^ Treg^26^. Thirdly, it has been reported that TNFα acted as a co-stimulator for TCR stimulation driven T cell activation^40^, stimulating Treg to respond. Last, mTNF/TNFR2 promotes IL-2 promoter activity and IL-2 mRNA stability, enhancing the sensitivity of Treg to a low level of IL-2 stimulation.

Taken together, our results suggest that exogenous TNFα promoted iTreg differentiation and function via TNFR2 signal pathway, implicating TNFR2 is a critical immune regulator. Thus, selective targeting on TNFR2 but not TNFα is more important since it has a limited distribution, making it less systemically toxic^41^. Moreover, understanding the precise effect of TNFα/TNFR2 on the development and function of iTreg will promote iTreg therapy for patients with autoimmune diseases in the future.

## Materials and methods

### Mice

All the mice were purchased from The Jackson Laboratory and Nanjing Animal Institute and bred and housed under specific pathogen-free conditions in Animal facilities of Hershey Medical Center, Penn State University and Sun Yat-sen University. All the mice used in this study are six to eight-week-old mice with C57BL/6 background. Foxp3-GFP reporter mice were backcrossed into TNFR1^−/−^ or TNFR2^−/−^ mice, yielding TNFR1^−/−^ or TNFR2^−/−^ Foxp3-reporter mice. All mice were housed and treated by National Institutes of Health guidelines for the use of experimental animals with the approval of Penn State University and Sun Yat-sen University Committees for the Use and Care of Animals.

### Antibodies and reagents

Naive CD4^+^ T cell isolation kit was purchased from Miltenyi Biotec (Cologne, Germany). Anti-CD3/CD28 coated beads were purchased from Gibco (New York, USA). RhIL-2, rhTGF-β, rmIL-6, and rmIL-12 were purchased from R&D Systems (Minneapolis, MN). Anti-TNFR1 PE, anti-TNFR2 PE, anti-Thy1.1 PercP/Cy5.5, anti-LAP APC, anti-PD-1 APC, anti-Nrp-1APC anti-GITR PE, anti- Ki-67 PE, anti-CD4 PercP/Cy5.5, anti-IFN-γ PE, anti-IL-17A APC, rmTNFα, anti-IL-4 antibody, anti-IFN-γ antibody, and CFSE were purchased from Biolegend (San Diego, CA). PMA, ionomycin, and cyclosporin A (CsA) were purchased from Calbiochem-EMD Millipore (Dormstadt, Germany). CFA was purchased from Sigma-Aldrich. Incomplete Freund's Adjuvant (IFA, Difco, MI, USA), killed *Mycobacterium tuberculosis* (strain H37Ra; Difco), and myelin oligodendrocyte glycoprotein (MOG, 35–55 peptide, (AnaSpec Inc, Fremont, CA) were also purchased.

### iTreg induction in vitro from naive CD4^+^ T cells

Naive CD4^+^CD62L^+^ T cells were isolated from spleen cells of C57BL/6 mice using naive CD4^+^ T cell isolation kit. The purity of the isolated cells was > 90%. Cells were subsequently cultured in 48-well plates and activated by anti-CD3/CD28 coated beads (five cells to one bead, Invitrogen), with recombinant human IL-2 (rhIL-2) 50 U/ml and rhTGF-β 2 ng/ml for three days. Recombinant mouse TNFα (rmTNFα) was added into wells accordingly. Foxp3 expression was determined by flow cytometry. The suppressive activity of these cells against effector T cell proliferation was examined with a standard in vitro suppressive assay as previously reported^22^.

### Treg expansion in vitro

Treg isolated from LNs of Foxp3-GFP reporter mice were marked with Foxp3-GFP and anti-CD4 PercP/Cy5.5 and sorted by flow cell sorter, the purity of Treg was > 98%. Sorted Treg were cultured with anti-CD3/CD28 coated beads (one cell to one bead) and rhIL-2 300 U/ml for three days.

### Th1 and Th17 cells differentiation from naive CD4^+^ T cells

Naive CD4^+^ T cells were stimulated with antibodies to CD3 (1 μg/ml) and CD28 (1 μg/ml), and anti-IL-4 monoclonal antibodies (5 μg/ml), in the presence or absence of TNFα (100 ng/ml). For Th1 cells differentiation, rmIL-12 (10 ng/ml) was added. For Th17 cells differentiation, rmIL-6 (10 ng/ml) and rhTGF-β (2 ng/ml), anti-IFN-γ (5 μg/ml) were added. Cells were harvested and stained with anti-IL-17A, anti-IFN-γ monoclonal antibody using the intracellular flow cytometry staining protocol.

### Treg suppressive function in vitro

Enriched effector T cells were labeled with 1uM CFSE 0.3 × 10^6^ cells /well of CFSE-labeled T cells were seeded in a flat-bottom 96-well plate in the medium with 0.3 × 10^6^ cells/well of APCs plus 0.025 μg/ml soluble anti-CD3 antibody. Generated iTreg at different ratios to Teffs were added in some wells. After 72 h incubation, the proliferation of CD8^+^ T cells was assessed by CFSE dilution using flow cytometry.

### Induction and assessment of colitis

0.6 × 10^6^ naive CD4^+^ T cells were purified from WT, TNFR1^−/−^, or TNFR2^−/−^ Foxp3-GFP reporter C57BL/6 mice and injected i.p. into Rag1^−/−^ mice. The weight of each mouse was noted every day. Three weeks and four weeks after transfer, the cells from spleens, mesenteric lymph nodes (mLNs) and colonic lamina proprias (cLP) were harvested from recipient mice^23^. The proportions of Foxp3^+^, IL-17A^+^, or IFN-γ^+^ T cells were determined by flow cytometry. To assess for colitis, the colon was subjected to H&E staining after four weeks. In other experiments, 0.6 × 10^6^ naive CD4^+^ cells isolated from Thy1.1^+^ mice were i.p. injected into Rag1^−/−^ mouse, similar numbers of nTregs isolated from thymus in Thy1.2^+^ mice or iTreg subset induced ex vivo from Thy1.2^+^ WT, TNFR1^−/−^, or TNFR2^−/−^ mice were i.v. injected into Rag1^−/−^ mice. The colitis was similarly assessed and frequency of Treg and Th1/Th17 cells was analyzed on the gate of either Thy1.2 (Treg) or Th1.1 (pathogenic cells) on the days indicated.

### Induction and treatment of EAE

EAE was induced in age-matched female mice with 300 μL of 300 μg MOG emulsified in CFA containing 8 mg/mL killed *Mycobacterium tuberculosis* via subcutaneous injection on day 0 and day 7, followed by the i.p. injection of 250 ng pertussis toxin (list biological laboratories) on day 0, day 2, day 7, day 9^13^. Clinical scores (0, no symptoms; 1, limp tail; 2, partial paralysis of hind limbs; 3, complete paralysis of hind limbs or partial hind and front limb paralysis; 4, tetraparalysis; 5, moribund) were recorded daily. The mean score was noted for each mouse every day.

For treatment, iTreg were injected intravenously on day 9 after the first immunization. Thirty days after the first immunization, the cells from spleens, inguinal LNs, brain, and spinal cord were harvested. The proportions of Foxp3^+^, IL-17A^+^, or IFN-γ^+^ T cells were determined by flow cytometry. Brain and spinal cord were subjected to H&E staining.

### Histopathology

For brain, the left anterior part was harvested. For spinal cord, 0.2 cm part of intumescentia lumbalis was harvested. The specimens were preserved in 10% buffered formalin, embedded in paraffin, blocked, sectioned, and stained with H&E. These global histological changes were evaluated by inflammatory cell infiltration, histomorphological change, and demyelination.

For colon in colitis, 1 mm colon from each mouse was removed and fixed in 10% buffered formalin, subsequently trimmed and spread out to render a transverse section. The specimens were processed, blocked, sectioned, and stained with H&E. The histological changes were evaluated for inflammatory cell infiltration, bowel wall thickness, ulceration, and edema.

### Statistical analysis

All of the results were calculated by GraphPad Prism 5.0 software (GraphPad Software, San Diego, CA) and presented as mean ± standard deviation (SD). Student’s *t*-test was used to assess statistical significance between two groups, and one-way ANOVA and/or non-parametric tests were used to assess statistical significance among multi-groups. *P* ≤ 0.05 were considered as a statistically significant difference.

## Supplementary information

Supplementary information
