# Supplementary material for: Differential roles of TNFα-TNFR1 and TNFα-TNFR2 in the differentiation and function of CD4+Foxp3+ induced Treg cells in vitro and in vivo periphery in autoimmune diseases
Source: Cell Death Dis. 2019 Jan 10;10(1):27. doi: 10.1038/s41419-018-1266-6 (PMC6328545; doi:10.1038/s41419-018-1266-6)
Supplement: Supplementary file 1 — Supplementary information [file 41419_2018_1266_MOESM1_ESM.doc]

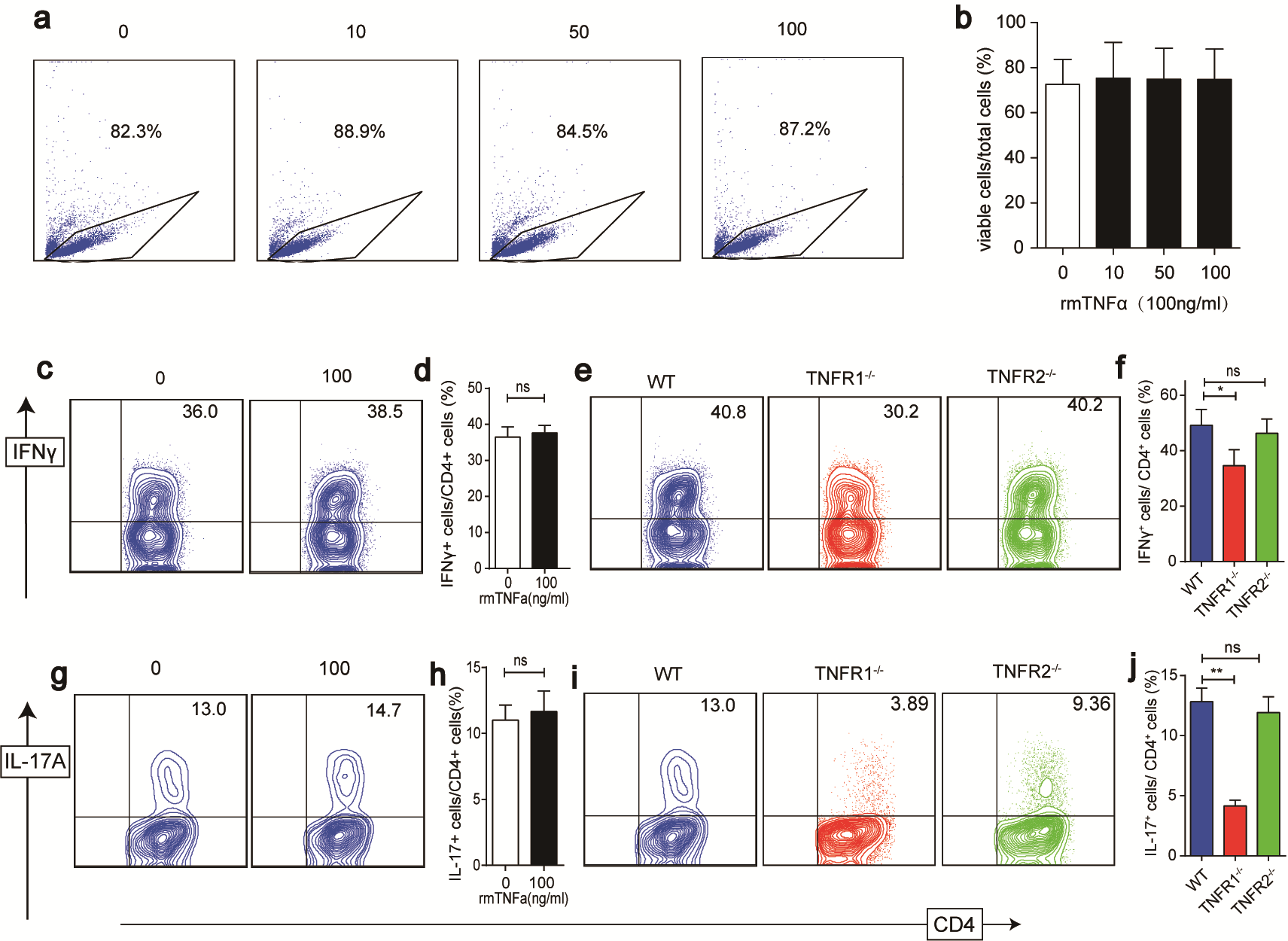


**Supplement 1. The effect of rmTNFα on iTreg stability and Th1 and Th17 differentiation *in vitro* and The effect of TNFR on Th1 and Th17 differentiation *in vivo.* (a, b)** Naive CD4^+^CD62L^+^ T cells isolated from WT mice were induced into iTregs as standard assay with different doses of rmTNFα. The viability of each group was detected by FACS. **(c, d, j, h)** Naive CD4^+^ T cells from WT mice were induced to differentiate to Th1, Th17 cells with or without TNFα exposure *in vitro.* **(e, f, i, j)** Naive CD4^+^ T cells derived from WT, TNFR1^-/-^ mice and TNFR2^-/-^ mice were differentiated to Th1and Th17 cells. *, *P* ≤ 0.05; **, *P* ≤ 0.01. Representative data is from five independent experiments.


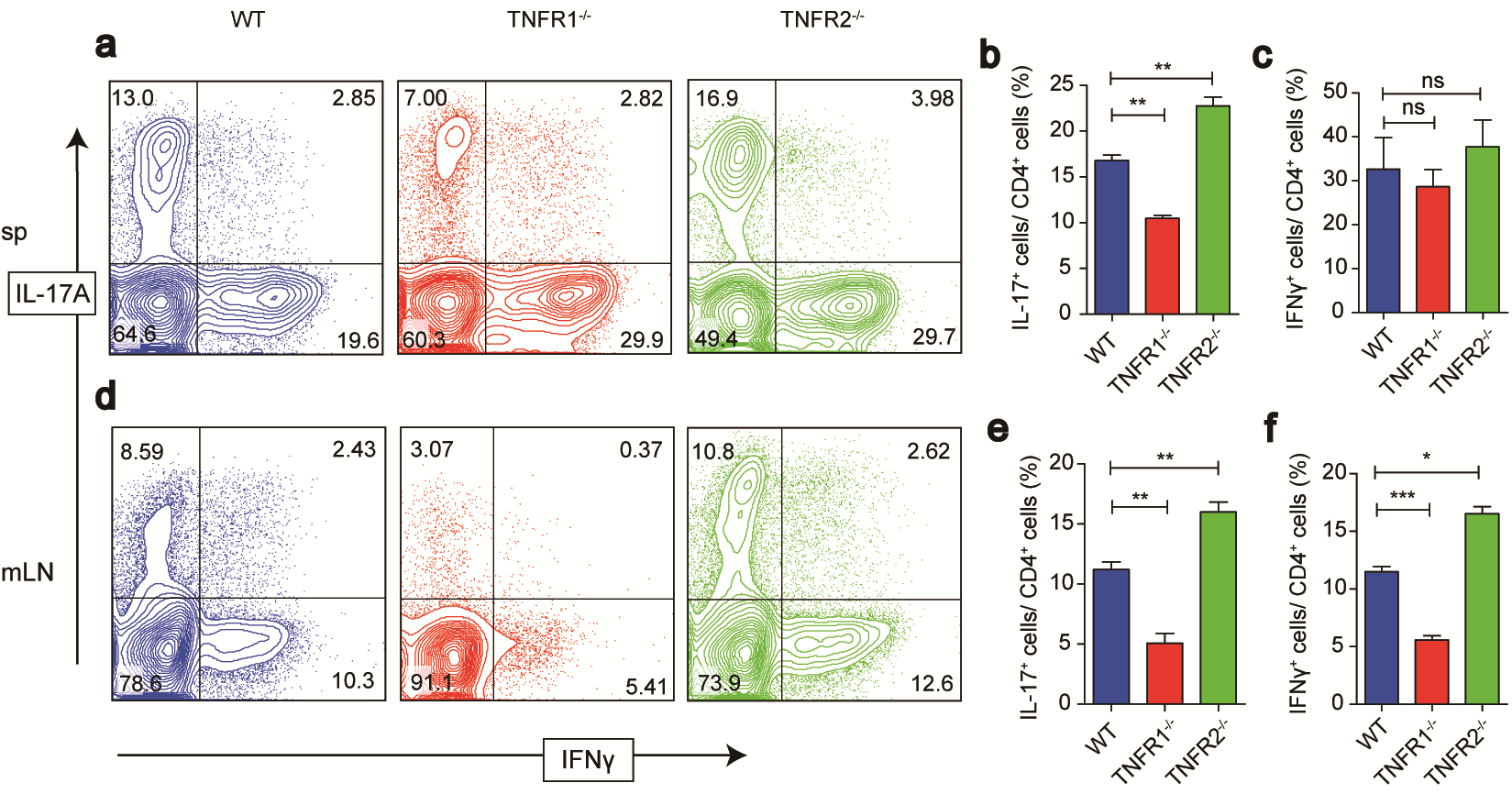


**Supplement 2. The effect of TNFR1 and TNFR2 on Th1 and Th17 cells differentiation *in vivo.* (a~f)** Naive CD4^+^ T cells isolated from WT, TNFR1^-/-^, TNFR2^-/-^ mice were injected into Rag1^-/-^ mice [intraperitoneal](../../../../../SUJUAN%20YANG/AppData/Local/youdao/dict/Application/7.2.0.0703/resultui/dict/)ly. 28 days after cell transfer, the recipient mice were sacrificed, spleens, mLNs were harvested and the proportions of IL-17^+^ or IFN-γ^+^ T cells were determined by FACS. *, *P* ≤ 0.05; **, *P* ≤ 0.01; ***, *P* ≤ 0.001. Representative data is from six independent experiments.


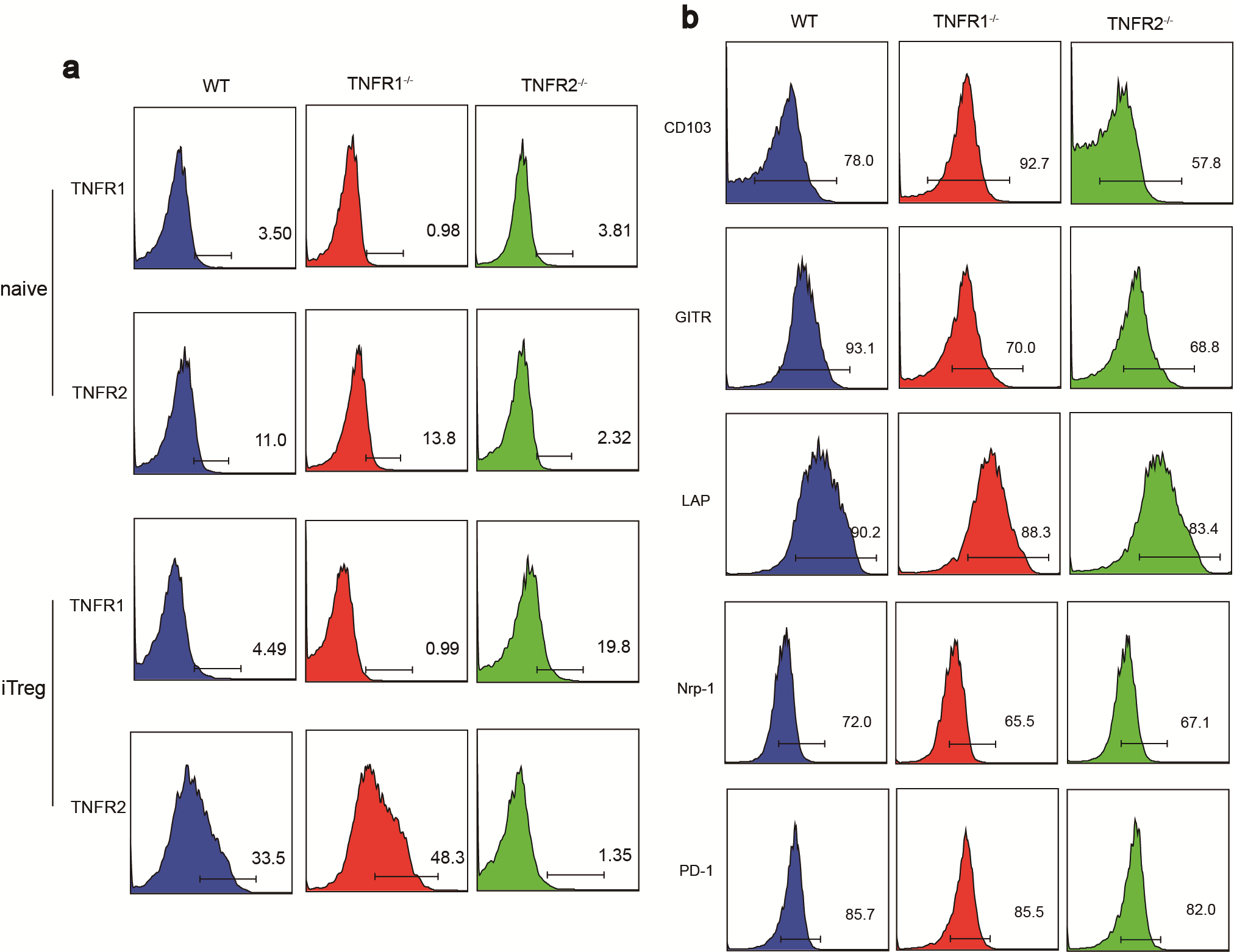


**Supplement 3**. (a) The level of TNFR1 and TNFR2 expression on naive CD4+ T cells and iTreg induced in vitro. Naive CD4+ T cells isolated from WT, TNFR1-/- and TNFR2-/-mice were induced into iTreg as standard assay. Expression of TNFR1 and TNFR2 on the surface of WT, TNFR1-/- and TNFR2-/- iTregs was detected by FACS. **(b)** The relations between TNFR1 or TNFR2 and iTreg phenotype. Naive CD4+ T cells isolated from WT, TNFR1-/- and TNFR2-/- mice were induced into iTregs as standard assay. Expression of TNFR1 and TNFR2 on the surface of WT, TNFR1-/- and TNFR2-/- iTregs was detected. Expression of CD103, GITR, LAP, Nrp-1 and PD-1 on the surface of WT, TNFR1-/- and TNFR2-/- iTregs was detected by FACS. Representative data is from five independent experiments.


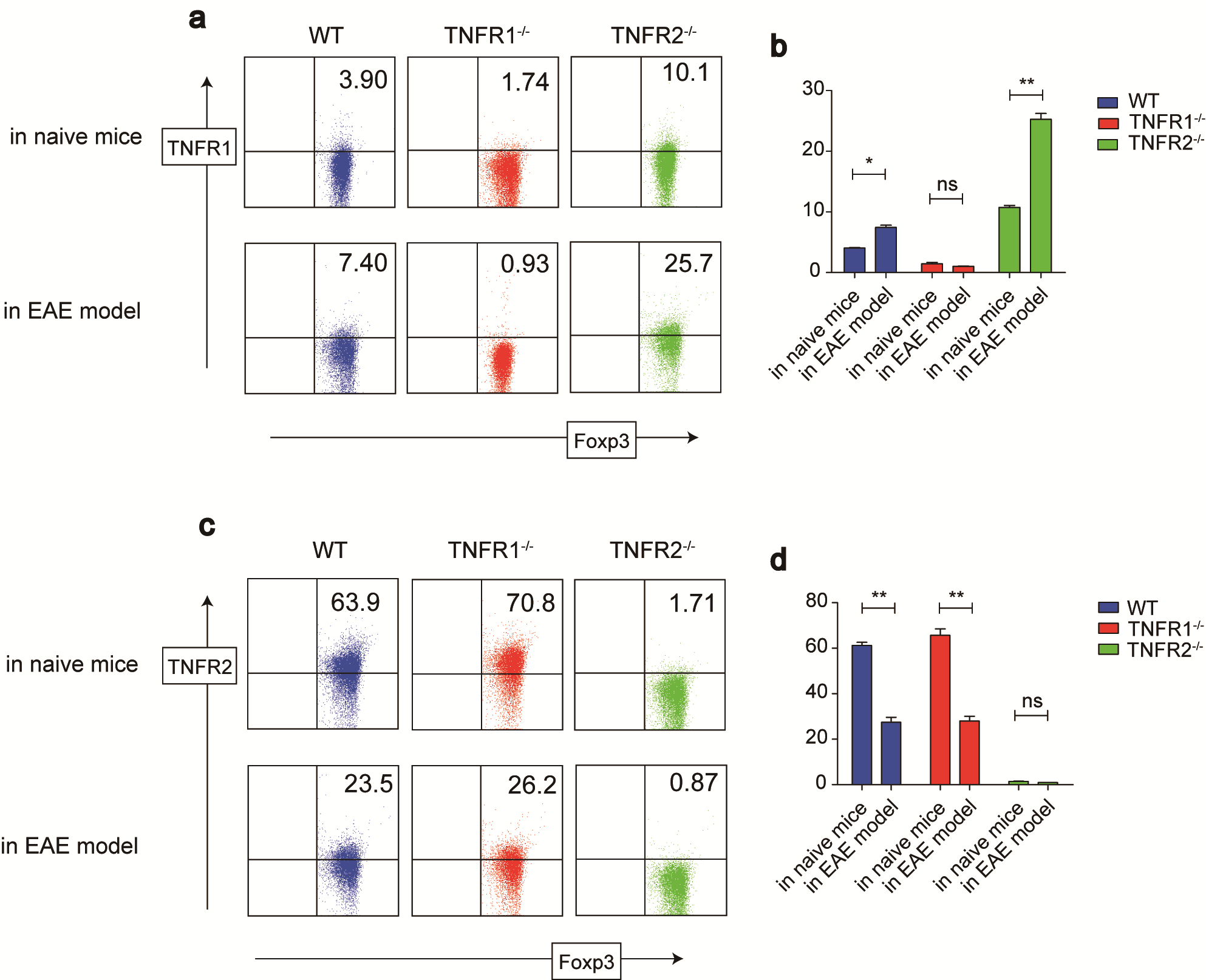


**Supplement 4**. TNFR1 and TNFR2 expression on nTreg from naive mice and EAE model. nTreg were isolated from naive mice and EAE model of WT, TNFR1^-/-^ and TNFR2^-/-^ mice. The level of TNFR1 and TNFR2 on nTreg were detected by FACS.*, *P* ≤ 0.05; **, *P* ≤ 0.01, error bars denote SD. Representative data is from six independent experiments.


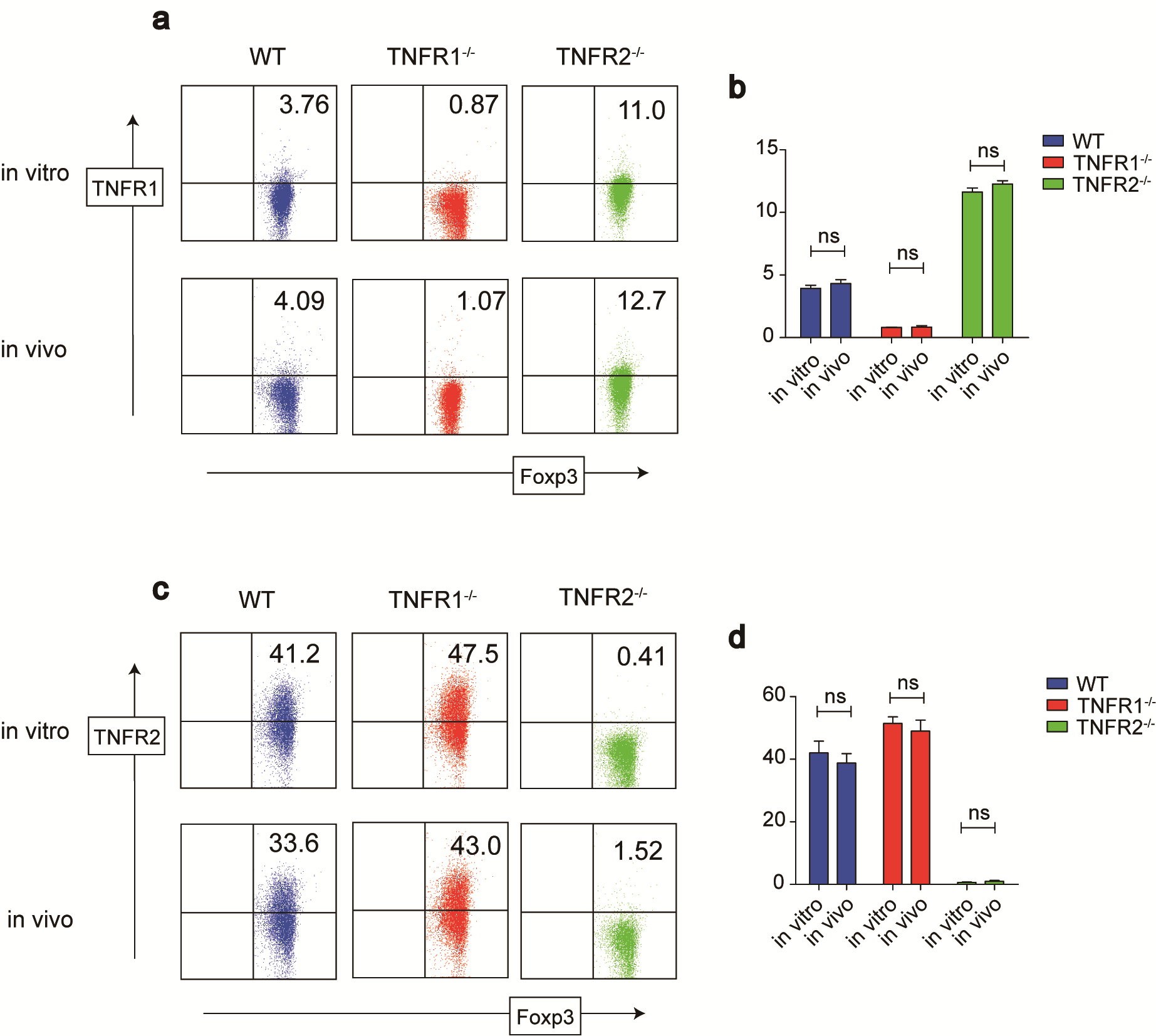


**Supplement 5**. TNFR1 and TNFR2 expression on iTregs induced from naive mice and colitis model. iTregs were induced *in vitro* from naive mice and *in vivo* from colitis model of WT, TNFR1^-/-^ and TNFR2^-/-^ mice. The level of TNFR1 and TNFR2 on iTregs were detected by FACS.*, error bars denote SD. Representative data is from six independent experiments.
